# Supplementary material for: Community structure unveils the path multiplicity in complex networks
Source: Nat Commun. 2026 Mar 6;17:2283. doi: 10.1038/s41467-026-70369-4 (PMC12972315; doi:10.1038/s41467-026-70369-4)
Supplement: Supplementary file 1 — Supplementary Information [file 41467_2026_70369_MOESM1_ESM.pdf]

Supplementary Information

Community structure unveils the path multiplicity in complex networks

Ye Deng[1,2], Jun Wu[1,2,*], Xin Lu[3*], Petter Holme[4,5], Daqing Li[6,7*], Zengru Di[1,2,8], Guanrong Chen[9], Jürgen Kurths[10,11]

[1]Department of Systems Science, Faculty of Arts and Sciences, Beijing Normal University, Zhuhai 519087, China; [2]International Academic Center of Complex Systems, Beijing Normal University, Zhuhai 519087, China; [3]College of Systems Engineering, National University of Defense Technology, Changsha 410073, China; [4]Department of Computer Science, Aalto University, Espoo 02150, Finland; [5]Center for Computational Social Science, Kobe University, Kobe 657-8501, Japan; [6]School of Reliability and Systems Engineering, Beihang University, Beijing 100083, China; [7]Department of Science and Technology, Civil Aviation University of China, Tianjin 300300, China; [8]School of Systems Science, Beijing Normal University, Beijing 100875, China; [9]Department of Electrical Engineering, City University of Hong Kong, Hong Kong SAR, China; [10]Research Domain Complexity Science, Potsdam Institute for Climate Impact Research, Potsdam 14473, Germany; [11]Department of Physics, Humboldt-Universität zu Berlin, Berlin 12489, Germany

This PDF file includes:

Tables S1 to S2
Figs. S1 to S9

Table S1. Metadata of 140 real-world networks, where N is the number of nodes, n_c is the number of communities, $\langle k \rangle$ is the average degree, L is the average shortest path length, E_{glob} is the global efficiency, D is the diameter, r is the assortativity coefficient, C is the clustering coefficient, and k_s is the maximum k-core. For each real network, we analyze the largest connected component to avoid artifacts from disconnected components.

$No.$	$Name$	$RPMI$	PMI	N	n_c	$\langle k \rangle$	L	E_{glob}	D	r	C	k_s
1	bn-cat-mixed-species-brain-1	1.0756	5.7591	65	3	22.4615	1.6995	0.6671	3	-0.0254	0.6614	16
2	bn-macaque-rhesus-brain-2	0.9842	4.0379	91	2	12.7912	1.8681	0.5694	3	-0.7698	0.8601	11
3	bn-macaque-rhesus-cerebral-cortex-1	1.1219	8.0681	91	3	30.7912	1.6581	0.6710	3	-0.5534	0.7424	22
4	bn-macaque-rhesus-interareal-cortical-network-2	1.1343	14.1950	93	1	48.6452	1.4712	0.7644	2	-0.6957	0.8498	29
5	bn-mouse-kasthuri-graph-v4	7.9077	13.2047	987	21	3.1125	4.9136	0.2264	12	-0.2417	0	3
6	bn-mouse-brain-1	0.8874	27.9580	213	2	151.0704	1.2874	0.8563	2	-0.0672	0.7583	111
7	bn-mouse-retina-1	1.3096	29.4874	1076	4	168.7937	1.8613	0.5755	4	-0.1976	0.5906	121
8	bn-mouse-visual-cortex-1-2	1.4822	2.1108	29	3	3.0345	2.9409	0.4177	6	-0.4709	0.0494	2
9	aves-barn-swallow-contact-network	0.9912	2.0809	17	2	6.2353	1.7279	0.6759	4	-0.3103	0.5482	5
10	aves-barn-swallow-non-physical	0.8488	1.8529	17	2	14.3529	1.1029	0.9485	2	-0.1528	0.9290	13
11	aves-sparrow-social	1.1362	4.7888	52	4	17.4615	1.7489	0.6560	3	-0.0562	0.7187	13
12	aves-sparrow-social-2009	0.9034	3.2409	31	3	13.6129	1.8903	0.6781	4	0.2886	0.7693	12
13	aves-sparrow-social-2010	0.9755	3.9551	40	4	15.2500	1.6462	0.6893	3	-0.1159	0.7083	11
14	aves-sparrowlyon-flock-season2	0.9929	2.5770	43	3	6.9767	2.0177	0.5524	3	-0.6300	0.6711	8
15	aves-sparrowlyon-flock-season3	1.0288	3.4387	27	2	12.0741	1.6068	0.7203	3	0.0652	0.7445	10
16	aves-thornbill-farine	0.9397	3.2263	63	3	10.1587	1.8484	0.5799	3	-0.7506	0.8667	8
17	bio-CE-GT	2.5329	7.7224	878	22	7.2460	3.7349	0.2981	10	-0.1887	0.5889	9
18	bio-CE-LC	3.4967	4.7938	993	91	2.6183	7.9226	0.1464	22	-0.2597	0.0838	4
19	bio-CE-PG	0.6010	1.5815	734	3	5.6676	2.0862	0.4882	3	-0.5511	0.4940	9
20	bio-DM-HT	6.3013	10.8539	2831	243	3.2229	8.1390	0.1335	19	-0.1020	0.0099	11
21	bio-DM-LC	21.2030	44.6744	483	34	4.1284	6.6134	0.1864	18	-0.1873	0.1248	10
22	bio-DR-CX	1.4943	27.7228	3287	4	51.6818	2.7809	0.3858	7	0.3436	0.1956	95
23	bio-HS-HT	3.4721	13.7214	2499	50	10.9244	4.0626	0.2684	12	0.2919	0.1731	39
24	bio-SC-LC	4.3169	21.5007	1999	15	20.4582	3.2396	0.3333	7	0.1946	0.1665	48
25	bio-SC-TS	1.0518	4.5234	101	2	12.9307	1.8828	0.5626	3	-0.8830	0.8964	9
26	bio-celegans	1.3685	4.9195	453	8	8.9404	2.6638	0.4072	7	-0.2258	0.6465	10
27	bio-celegans-dir	1.3694	4.9195	453	8	8.9404	2.6638	0.4072	7	-0.2258	0.6465	10
28	bio-grid-mouse	6.6541	9.7835	791	66	2.7762	8.6630	0.1536	30	-0.2145	0.0469	6
29	bio-grid-plant	3.7729	8.2150	1272	137	4.2862	8.2944	0.1465	26	0.0014	0.1676	12
30	bio-grid-worm	3.2690	6.5846	3343	36	3.8510	4.3195	0.2508	13	-0.1692	0.0512	10
31	bio-yeast	1.8313	2.5769	1458	152	2.6722	6.8124	0.1638	19	-0.2095	0.0708	5
32	bio-yeast-protein-inter	1.8357	2.5769	1458	152	2.6722	6.8124	0.1638	19	-0.2095	0.0708	5
33	ca-AstroPh	3.4334	25.1806	17903	611	22.0044	4.1940	0.2553	14	0.2013	0.6328	56
34	ca-CSphd	1.5825	1.5309	1025	138	2.0351	11.7484	0.1055	28	-0.2532	0.0034	2
35	ca-CondMat	3.3680	11.8434	21363	979	8.5462	5.3522	0.1979	15	0.1253	0.6417	25
36	ca-Erdos992	2.8702	4.5561	4991	430	2.9766	5.5116	0.1927	14	-0.4531	0.0835	7
37	ca-GrQc	1.8685	5.4007	4158	494	6.4560	6.0494	0.1792	17	0.6392	0.5569	43
38	ca-HepPh	2.8059	21.8267	11204	206	20.9959	4.6727	0.2315	13	0.6295	0.6216	238
39	eco-everglades	1.1377	7.0286	69	3	25.5072	1.6360	0.6857	3	-0.2983	0.5521	20

Continued on next page

No.	*Name*	*RPMI*	*PMI*	*N*	n_c	$\langle k \rangle$	*L*	E_{glob}	*D*	*r*	*C*	k_s
40	eco-foodweb-baydry	1.4322	9.3675	128	4	32.9063	1.7724	0.6243	3	-0.1044	0.3346	24
41	eco-foodweb-baywet	1.4071	9.1694	128	4	32.4219	1.7763	0.6224	3	-0.1117	0.3346	23
42	eco-mangwet	1.0693	7.0692	97	3	29.8144	1.6929	0.6547	3	-0.1506	0.4683	23
43	eco-stmarks	0.9088	3.1181	54	4	12.9630	1.7610	0.6214	3	-0.2322	0.4128	10
44	ENZYMES-g118	1.7952	2.2674	95	11	2.5474	7.5684	0.1884	18	0.1012	0	2
45	ENZYMES-g196	1.0641	1.8327	50	6	3.4400	4.1200	0.3236	10	-0.0603	0.2116	3
46	ENZYMES-g198	1.1528	1.6505	55	6	2.8000	5.1461	0.2678	13	0.0590	0.1867	2
47	ENZYMES-g203	1.3057	2.3312	56	5	3.5714	4.7403	0.2911	11	0.2239	0.2774	3
48	ENZYMES-g209	1.3509	2.2888	57	6	3.5439	4.7976	0.2872	11	0.2284	0.2830	3
49	ENZYMES-g349	1.4856	2.6394	64	7	3.6875	5.3492	0.2774	12	-0.2252	0.2395	3
50	ENZYMES-g465	1.4310	2.6237	52	7	3.8077	4.4329	0.3126	9	0.0134	0.2462	3
51	ENZYMES-g484	1.4834	2.0243	60	5	2.6667	6.0068	0.2392	13	0.0585	0.1528	2
52	ENZYMES-g501	1.4527	1.6732	66	8	2.4242	7.1403	0.2100	18	0.4907	0.0101	3
53	ENZYMES-g532	1.4661	2.2658	74	7	3.2432	4.7516	0.2753	12	0.2030	0.0921	3
54	ENZYMES-g541	1.4041	1.7804	28	6	2.6429	3.8016	0.3509	8	-0.0735	0.0560	2
55	econ-mahindas	6.5349	30.4553	1258	10	11.9444	3.5743	0.3052	8	-0.0622	0.0613	40
56	econ-poli	4.2807	4.8481	2343	216	2.2766	10.2002	0.1174	27	-0.3352	0.0674	5
57	econ-wm3	1.5775	10.2173	257	5	18.5136	2.6147	0.4391	6	-0.0648	0.2653	33
58	ia-email-univ	1.7528	6.7378	1133	14	9.6222	3.6060	0.2999	8	0.0782	0.2202	11
59	ia-hospital-ward-proximity	0.9573	7.3337	75	3	30.3733	1.5978	0.7038	3	-0.1808	0.6403	22
60	ia-hospital-ward-proximity-attr	0.9561	7.3337	75	3	30.3733	1.5978	0.7038	3	-0.1808	0.6403	22
61	ia-infect-hyper	0.9514	8.6923	113	4	38.8673	1.6563	0.6730	3	-0.1226	0.5348	28
62	ia-reality	4.4410	5.0597	6809	337	2.2558	4.2136	0.2455	8	-0.6753	0.0178	5
63	ia-southernwomen	0.8721	1.9608	18	2	7.1111	1.6209	0.7026	3	-0.3641	0.7619	6
64	ia-workplace-contacts	1.2810	5.0535	92	4	16.4130	1.9644	0.5661	3	-0.0586	0.4260	11
65	power-1138-bus	2.1787	2.9435	1138	56	2.5624	12.7237	0.0969	31	-0.0801	0.0866	4
66	power-494-bus	1.8915	2.2946	494	29	2.3725	10.4701	0.1184	26	-0.0739	0.0420	2
67	power-662-bus	1.6901	2.4478	662	27	2.7372	10.2445	0.1183	25	-0.0696	0.0462	4
68	power-685-bus	3.7258	7.2922	685	17	3.7431	12.4221	0.1085	26	0.1812	0.1725	5
69	power-US-Grid	4.9971	6.9937	4941	141	2.6691	18.9892	0.0629	46	0.0035	0.0801	5
70	power-bcspwr09	3.0977	4.5706	1723	76	2.7789	15.4879	0.0812	37	-0.1001	0.0759	3
71	power-bcspwr10	3.8988	6.5410	5300	100	3.1211	20.8466	0.0583	49	-0.0527	0.0880	4
72	copresence-InVS13	0.6581	6.2795	95	2	82.4211	1.1232	0.9384	2	-0.0268	0.9278	78
73	hospital-ward-proximity	0.9636	7.3337	75	3	30.3733	1.5978	0.7038	3	-0.1808	0.6403	22
74	infect-hyper	0.9573	8.6923	113	4	38.8673	1.6563	0.6730	3	-0.1226	0.5348	28
75	primary-school-proximity	1.1019	15.6916	242	4	68.7355	1.7325	0.6397	3	0.1183	0.5255	47
76	road-City-of-Oldenburg	2.1546	2.4921	6105	120	2.3027	40.6904	0.0305	104	0.0554	0.0108	2
77	road-beijing	12.7863	18.6076	5036	101	2.7347	36.9239	0.0346	89	0.2664	0.0244	2
78	road-chesapeake	0.9422	2.7193	39	3	8.7180	1.8354	0.6039	3	-0.3758	0.4502	6
79	road-euroroad	2.3307	3.0365	1039	25	2.5120	18.3951	0.0772	62	0.0900	0.0189	2
80	road-minnesota	7.2982	9.5668	2640	40	2.5015	35.3491	0.0401	99	-0.1866	0.0160	2
81	tech-routers-rf	2.3901	6.7771	2113	135	6.2773	4.6074	0.2381	12	0.0192	0.2464	15
82	email-dnc	2.5819	6.0927	1833	18	4.7638	3.3695	0.3190	8	-0.3088	0.2157	17
83	email-univ	1.7744	6.7378	1133	14	9.6222	3.6060	0.2999	8	0.0782	0.2202	11
84	inf-USAir97	1.3100	5.5779	332	4	12.8072	2.7381	0.4059	6	-0.2079	0.6252	26
85	inf-euroroad	2.3747	3.0365	1039	25	2.5120	18.3951	0.0772	62	0.0900	0.0189	2
86	inf-openflights	5.6518	21.8166	2905	29	10.7711	4.0971	0.2669	14	0.0489	0.4555	28
87	inf-power	4.9972	6.9937	4941	141	2.6691	18.9892	0.0629	46	0.0035	0.0801	5
88	london-overground	1.5239	1.7782	369	21	2.3306	13.7327	0.1014	39	0.1370	0.0294	2

Continued on next page

No.	*Name*	*RPMI*	*PMI*	*N*	n_c	$\langle k \rangle$	*L*	E_{glob}	*D*	*r*	*C*	k_s
89	london-underground	1.5184	1.7520	271	19	2.3026	13.9633	0.1045	39	0.1380	0.0311	2
90	web-EPA	3.7062	7.9616	4253	113	4.1839	4.5003	0.2356	10	-0.3041	0.0714	6
91	web-indochina-2004	8.4246	28.5535	11358	1105	8.3828	6.4182	0.1773	27	0.1246	0.7099	49
92	web-spam	3.2229	18.8184	4767	28	15.6807	3.7935	0.2908	12	0.00004	0.2860	35
93	web-webbase-2001	35.7642	60.9623	16062	1327	3.1868	10.2092	0.1246	23	-0.0985	0.2243	32
94	soc-dolphins	1.3573	2.9995	62	4	5.1290	3.3570	0.3792	8	-0.0436	0.2590	4
95	soc-firm-hi-tech	0.9853	2.2311	33	3	5.5152	2.3598	0.5081	5	-0.0874	0.4533	5
96	soc-hamsterster	5.4328	29.9968	2000	28	16.0970	3.5890	0.3056	10	0.0227	0.5400	24
97	soc-karate	1.3983	2.7736	34	4	4.5882	2.4082	0.4920	5	-0.4756	0.5706	4
98	soc-tribes	0.9268	2.1833	16	3	7.2500	1.5417	0.7375	3	0.0499	0.5392	5
99	soc-wiki-Vote	2.4204	7.0649	889	12	6.5557	4.0962	0.2735	13	-0.0288	0.1528	9
100	fb-pages-food	3.0802	9.0002	620	62	6.7452	5.0887	0.2417	17	-0.0322	0.3309	11
101	fb-pages-politician	4.0911	18.9960	5908	205	14.1185	4.6641	0.2319	14	0.0182	0.3851	31
102	fb-pages-public-figure	5.5812	25.4414	11565	295	11.5933	4.6230	0.2322	15	0.2020	0.1793	42
103	fb-pages-tvshow	4.9838	18.2799	3892	508	8.8587	6.2759	0.1793	20	0.5605	0.3737	56
104	socfb-Reed98	1.3020	16.6017	962	4	39.1102	2.4615	0.4417	6	0.0234	0.3184	34
105	socfb-nips-ego	1.5803	1.5279	2888	17	2.0644	3.8674	0.2934	9	-0.6682	0.0272	3
106	rt-retweet	1.3223	1.5292	96	10	2.4375	4.3105	0.2765	10	-0.1792	0.0607	3
107	rt-twitter-copen	1.8398	2.6331	761	42	2.7043	5.3528	0.2086	14	-0.0992	0.0759	4
108	rt-assad	3.4163	4.6820	2139	50	2.6050	5.2070	0.2124	14	-0.2639	0.0143	5
109	rt-damascus	4.6052	6.0862	3052	399	2.5354	5.1213	0.2180	13	-0.1624	0.0105	5
110	rt-obama	5.5761	5.7018	3212	254	2.1308	7.3120	0.1591	18	-0.2768	0.0037	3
111	rt-occupy	3.2230	4.1108	3225	268	2.4428	5.7192	0.1990	24	-0.1420	0.0114	4
112	rt-voteonedirection	12.5404	13.1401	2280	66	2.1614	4.7499	0.2488	16	-0.3924	0.0019	3
113	C125-9	0.9957	10.9378	125	2	111.4080	1.1015	0.9492	2	-0.0221	0.8990	102
114	MANN-a9	1.0522	3.7636	45	1	40.8000	1.0727	0.9636	2	0.0049	0.9232	40
115	brock200-1	1.0031	28.7842	200	3	148.3400	1.2546	0.8727	2	-0.0039	0.7451	134
116	gen200-p0-9-44	0.9929	16.8990	200	2	179.1000	1.1000	0.9500	2	-0.0146	0.9003	167
117	johnson8-4-4	1.0894	10.9710	70	5	53	1.2319	0.8841	2	0	0.7446	53
118	san200-0-9-1	0.9804	16.6245	200	2	179.1000	1.1000	0.9500	2	0.0406	0.9018	162
119	bio-celegansneural	1.4051	7.1399	297	4	14.4646	2.4553	0.4448	5	-0.1632	0.2924	10
120	chesapeake	0.9266	2.7193	39	3	8.7180	1.8354	0.6039	3	-0.3758	0.4502	6
121	delaunay-n10	23.1244	63.3303	1024	14	5.9688	10.3155	0.1225	20	-0.1153	0.4356	4
122	delaunay-n11	63.5916	174.7036	2048	18	5.9834	13.3452	0.0943	26	-0.1014	0.4365	4
123	insecta-ant-trophallaxis-colony1	1.0482	3.4683	41	3	12.4878	1.7415	0.6472	3	0.0032	0.3859	9
124	insecta-ant-trophallaxis-colony2	1.0662	3.4939	39	2	12.5641	1.7638	0.6498	4	-0.1544	0.4227	10
125	mammalia-raccoon-proximity	0.7317	2.5290	24	2	18.8333	1.1812	0.9094	2	-0.1331	0.9032	17
126	reptilia-tortoise-network-bsv	1.3165	3.2521	121	9	5.5372	3.4561	0.3417	9	0.1997	0.3674	6
127	reptilia-tortoise-network-cs	1.0989	2.6022	31	5	6	2.4022	0.5189	6	-0.0018	0.4635	5
128	reptilia-tortoise-network-fi	1.7979	3.6190	496	54	3.9677	7.9334	0.1552	21	0.3451	0.3365	8
129	reptilia-tortoise-network-hw	1.4844	1.2667	6	2	2	1.7333	0.6778	3	-0.7333	0	2
130	reptilia-tortoise-network-lm	1.0986	2.2085	41	4	4.8780	2.6439	0.4534	6	0.0407	0.3793	5
131	reptilia-tortoise-network-pv	1.0692	2.0606	22	3	4.4545	2.4589	0.5183	6	-0.1788	0.5164	5
132	DD199	62.5752	141.2739	841	36	4.5232	16.0853	0.0877	59	0.3298	0.4678	4
133	DD68	7.9454	20.5165	775	24	5.4013	12.1167	0.1297	41	0.4315	0.4391	5

Continued on next page

$No.$	$Name$	$RPMI$	PMI	N	n_c	$\langle k \rangle$	L	E_{glob}	D	r	C	k_s
134	DD687	4.7037	14.3214	725	16	7.1724	6.4497	0.1846	17	0.3577	0.4746	5
135	citeseer	3.5091	6.4720	2110	165	3.4768	9.3105	0.1275	28	0.0071	0.1711	7
136	cora	2.4179	5.0657	2485	149	4.0797	6.3110	0.1776	19	-0.0714	0.2376	4
137	gene	1.7007	3.1640	814	67	3.5283	7.0083	0.1654	22	0.1356	0.2231	12
138	internet-industry-partnerships	1.3454	3.5067	219	6	5.7534	2.9822	0.3701	6	-0.2168	0.1762	6
139	webkb-wisc	2.1146	3.8737	251	13	3.5857	3.2600	0.3473	8	-0.1934	0.2077	4
140	tech-WHOIS	3.5623	17.3702	7476	102	15.2335	3.5433	0.2994	8	-0.0425	0.4889	88

Table S2. Community counts of 140 real-world networks under multiple community detection algorithms. Columns correspond to the number of communities detected by each algorithm.

No.	Name	N	Leading Eigenvector	Walktrap	Leiden	Label Propagation	Infomap	Louvain
1	bn-cat-mixed-species__brain__1	65	3	4	4	1	1	4
2	bn-macaque-rhesus__brain__2	91	2	1	3	1	1	4
3	bn-macaque-rhesus__cerebral-cortex__1	91	3	1	3	1	1	3
4	bn-macaque-rhesus__interareal-cortical-network__2	93	1	1	1	1	1	1
5	bn-mouse-kasthuri__graph__v4	987	21	117	20	82	109	22
6	bn-mouse__brain__1	213	2	2	3	1	1	3
7	bn-mouse__retina__1	1076	4	10	5	1	1	5
8	bn-mouse__visual-cortex__1-2	29	3	6	3	3	4	5
9	aves-barn-swallow-contact-network	17	2	4	3	1	1	2
10	aves-barn-swallow-non-physical	17	2	2	2	1	1	2
11	aves-sparrow-social	52	4	5	4	1	1	4
12	aves-sparrow-social-2009	31	3	4	3	3	2	3
13	aves-sparrow-social-2010	40	4	3	3	1	1	3
14	aves-sparrowlyon-flock-season2	43	3	7	3	1	1	3
15	aves-sparrowlyon-flock-season3	27	2	2	2	1	1	2
16	aves-thornbill-farine	63	3	1	3	1	1	3
17	bio-CE-GT	878	22	61	17	29	51	17
18	bio-CE-LC	993	91	59	27	95	104	27
19	bio-CE-PG	734	3	1	4	2	1	4
20	bio-DM-HT	2831	243	336	45	292	310	43
21	bio-DM-LC	483	34	58	18	47	52	18
22	bio-DR-CX	3287	4	170	7	2	143	5
23	bio-HS-HT	2499	50	293	20	49	204	19
24	bio-SC-LC	1999	15	276	10	6	106	10
25	bio-SC-TS	101	2	1	2	1	1	3
26	bio-celegans	453	8	35	9	6	36	10
27	bio-celegans-dir	453	8	35	10	6	37	11
28	bio-grid-mouse	791	66	92	26	76	84	26
29	bio-grid-plant	1272	137	104	28	97	102	27
30	bio-grid-worm	3343	36	269	27	103	277	31
31	bio-yeast	1458	152	174	32	172	175	31
32	bio-yeast-protein-inter	1458	152	174	32	174	180	32

Continued on next page

No.	Name	N	Leading Eigenvector	Walktrap	Leiden	Label Propagation	Infomap	Louvain
33	ca-AstroPh	17903	611	2264	38	347	898	37
34	ca-CSphd	1025	138	66	33	136	137	32
35	ca-CondMat	21363	979	2306	56	1552	1337	54
36	ca-Erdos992	4991	430	197	36	330	296	33
37	ca-GrQc	4158	494	448	42	375	367	40
38	ca-HepPh	11204	206	2631	43	368	758	39
39	eco-everglades	69	3	2	3	1	1	4
40	eco-foodweb-baydry	128	4	2	4	1	1	4
41	eco-foodweb-baywet	128	4	2	4	1	1	5
42	eco-mangwet	97	3	2	4	1	1	4
43	eco-stmarks	54	4	8	4	1	1	4
44	ENZYMES_g118	95	11	13	9	16	14	9
45	ENZYMES_g196	50	6	5	5	9	7	5
46	ENZYMES_g198	55	6	9	8	7	10	6
47	ENZYMES_g203	56	5	6	5	7	9	5
48	ENZYMES_g209	57	6	6	6	6	10	6
49	ENZYMES_g349	64	7	6	6	8	9	6
50	ENZYMES_g465	52	7	8	7	9	9	7
51	ENZYMES_g484	60	5	9	7	4	10	7
52	ENZYMES_g501	66	8	8	9	16	11	9
53	ENZYMES_g532	74	7	12	6	13	12	5
54	ENZYMES_g541	28	6	5	5	5	5	5
55	econ-mahindas	1258	10	195	8	3	120	9
56	econ-poli	2343	216	88	45	216	208	45
57	econ-wm3	257	5	8	6	4	19	4
58	ia-email-univ	1133	14	49	11	15	77	11
59	ia-hospital-ward-proximity	75	3	3	4	1	1	4
60	ia-hospital-ward-proximity-attr	75	3	3	4	1	1	5
61	ia-infect-hyper	113	4	5	5	1	1	6
62	ia-reality	6809	337	68	45	81	91	43
63	ia-southernwomen	18	2	2	2	2	1	2
64	ia-workplace-contacts	92	4	4	4	1	4	4
65	power-1138-bus	1138	56	87	27	133	134	25
66	power-494-bus	494	29	37	21	67	69	19
67	power-662-bus	662	27	42	19	60	76	19
68	power-685-bus	685	17	21	17	50	61	16
69	power-US-Grid	4941	141	364	43	497	486	39
70	power-bcspwr09	1723	76	50	25	174	178	25
71	power-bcspwr10	5300	100	69	37	375	385	34
72	copresence-InVS13	95	2	2	2	1	1	2
73	hospital-ward-proximity	75	3	3	4	1	1	4
74	infect-hyper	113	4	5	5	1	1	6
75	primary-school-proximity	242	4	5	6	2	1	6
76	road City of Oldenburg	6105	120	177	55	526	630	55
77	road beijing	5036	101	92	41	444	448	45
78	road-chesapeake	39	3	2	3	1	1	3
79	road-euroroad	1039	25	67	25	90	134	22

Continued on next page

No.	Name	N	Leading Eigenvector	Walktrap	Leiden	Label Propagation	Infomap	Louvain
80	road-minnesota	2640	40	46	32	199	267	31
81	tech-routers-rf	2113	135	278	22	97	176	20
82	email-dnc	1833	18	67	17	32	78	13
83	email-univ	1133	14	49	9	5	72	13
84	inf-USAir97	332	4	27	6	7	20	7
85	inf-euroroad	1039	25	67	22	108	125	22
86	inf-openflights	2905	29	194	25	63	159	27
87	inf-power	4941	141	364	42	504	490	40
88	london_overground	369	21	45	17	55	56	19
89	london_underground	271	19	31	16	42	43	17
90	web-EPA	4253	113	206	27	112	207	30
91	web-indochina-2004	11358	1105	996	95	847	929	71
92	web-spam	4767	28	595	24	56	308	32
93	web-webbase-2001	16062	1327	387	81	682	537	71
94	soc-dolphins	62	4	4	5	5	6	5
95	soc-firm-hi-tech	33	3	5	4	2	4	3
96	soc-hamsterster	2000	28	202	28	57	155	22
97	soc-karate	34	4	5	4	2	3	4
98	soc-tribes	16	3	3	3	1	1	3
99	soc-wiki-Vote	889	12	42	9	7	74	10
100	fb-pages-food	620	62	54	18	29	54	18
101	fb-pages-politician	5908	205	260	29	141	241	30
102	fb-pages-public-figure	11565	295	889	42	179	724	34
103	fb-pages-tvshow	3892	508	443	49	238	290	46
104	socfb-Reed98	962	4	78	6	1	19	6
105	socfb-nips-ego	2888	17	6	8	10	11	8
106	rt-retweet	96	10	10	8	14	16	8
107	rt-twitter-copen	761	42	108	21	82	100	20
108	rt_assad	2139	50	217	30	139	190	28
109	rt_damascus	3052	399	90	27	149	199	29
110	rt_obama	3212	254	102	40	192	236	39
111	rt_occupy	3225	268	277	45	270	309	40
112	rt_voteonedirection	2280	66	105	32	69	123	32
113	C125-9	125	2	2	3	1	1	3
114	MANN-a9	45	1	1	3	1	1	3
115	brock200-1	200	3	3	5	1	1	5
116	gen200-p0-9-44	200	2	1	4	1	1	4
117	johnson8-4-4	70	5	1	5	1	1	5
118	san200-0-9-1	200	2	2	2	1	1	2
119	bio-celegansneural	297	4	22	6	1	7	6
120	chesapeake	39	3	2	3	1	1	4
121	delaunay_n10	1024	14	14	16	44	58	13
122	delaunay_n11	2048	18	23	18	109	110	19
123	insecta-ant-trophallaxis-colony1	41	3	6	4	1	1	4
124	insecta-ant-trophallaxis-colony2	39	2	6	4	1	1	5
125	mammalia-raccoon-proximity	24	2	2	2	1	1	2

Continued on next page

No.	Name	N	Leading Eigenvector	Walktrap	Leiden	Label Propagation	Infomap	Louvain
126	reptilia-tortoise-network-bsv	121	9	16	9	5	14	7
127	reptilia-tortoise-network-cs	31	5	5	4	1	3	4
128	reptilia-tortoise-network-fi	496	54	39	23	56	62	21
129	reptilia-tortoise-network-hw	6	2	2	2	1	1	2
130	reptilia-tortoise-network-lm	41	4	7	5	2	7	5
131	reptilia-tortoise-network-pv	22	3	4	3	3	3	4
132	DD199	841	36	41	25	92	87	24
133	DD68	775	24	42	21	65	68	21
134	DD687	725	16	30	16	63	58	16
135	citeseer	2110	165	270	40	197	243	36
136	cora	2485	149	163	26	169	242	30
137	gene	814	67	91	22	85	105	23
138	internet-industry-partnerships	219	6	32	8	2	23	7
139	webkb-wisc	251	13	31	12	10	35	12
140	tech-WHOIS	7476	102	453	24	43	297	19

Leading Eigenvector method, ρ$_p$= 0.2304, ρ$_s$=0.8497, QCR=0.9857

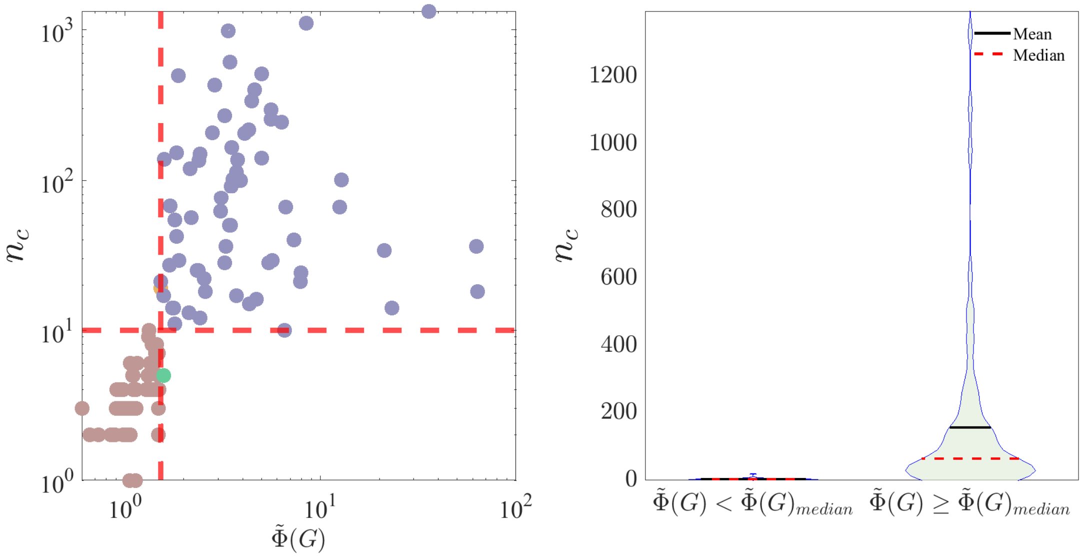

Fig. S1. Correlations between the relative path multiplicity and community number detected by the Leading Eigenvector method in 140 real-world networks. The metadata and results for all 140 networks are provided in Table S1. Each panel presents a scatter plot on the left, illustrating the correlation between the relative path multiplicity and community number (detected by Leading Eigenvector method) based on the given metric and their RPMI values. The networks are categorized into four quadrants, divided by the median values of community number n_c and the RPMI $\tilde{\Phi}(G)$. The right-hand violin plots show the distribution, mean and median values of community number, which are divided into two parts based on the median value of the RPMI. If there are more overlap between two violin subplots, it suggests a weaker correlation between community number and the RPMI. All community detection packages/functions and parameter settings used in this study, together with brief documentation, are available at https://github.com/gituserbnu/path-multiplicity.git.

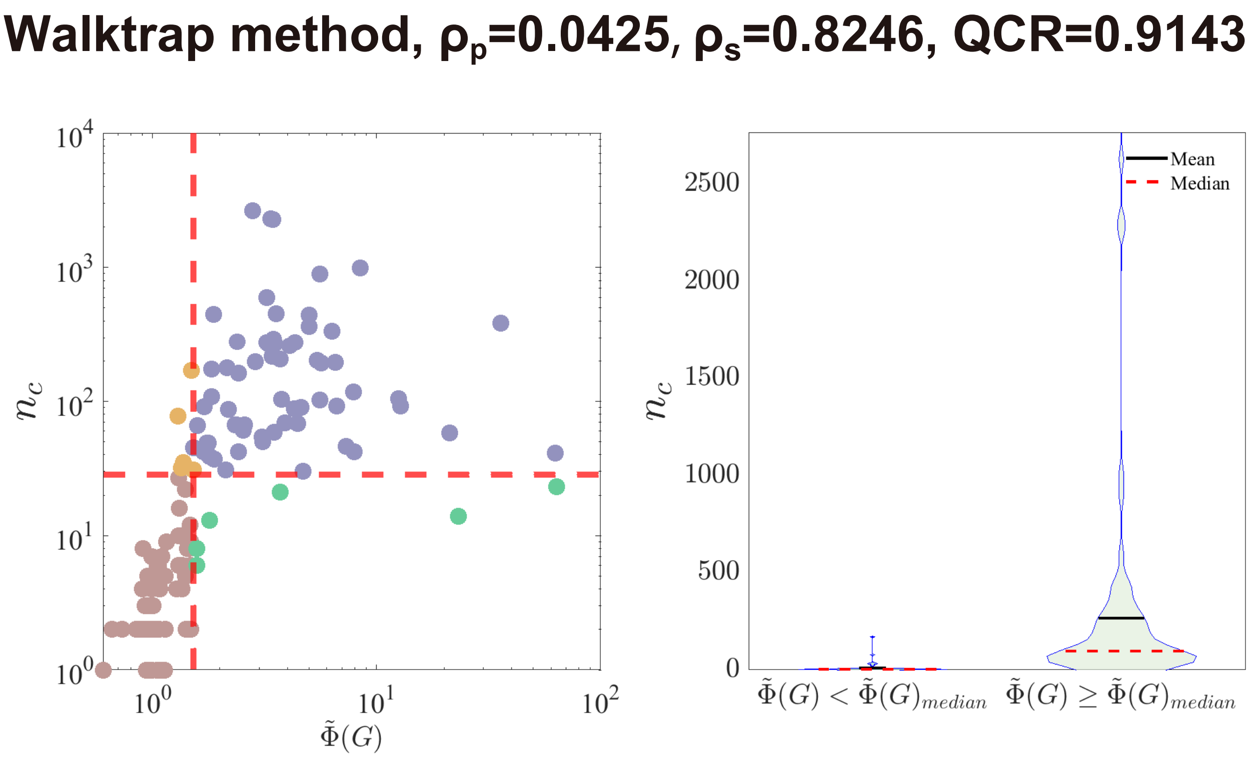

Fig. S2. Correlations between the relative path multiplicity and community number detected by the Walktrap method in 140 real-world networks. The metadata and results for all 140 networks are provided in Table S1. Each panel presents a scatter plot on the left, illustrating the correlation between the relative path multiplicity and community number (detected by Walktrap method) based on the given metric and their RPMI values. The networks are categorized into four quadrants, divided by the median values of community number n_c and the RPMI $\tilde{\Phi}(G)$. The right-hand violin plots show the distribution, mean and median values of community number, which are divided into two parts based on the median value of the RPMI. If there are more overlap between two violin subplots, it suggests a weaker correlation between community number and the RPMI. All community detection packages/functions and parameter settings used in this study, together with brief documentation, are available at https://github.com/gituserbnu/path-multiplicity.git.

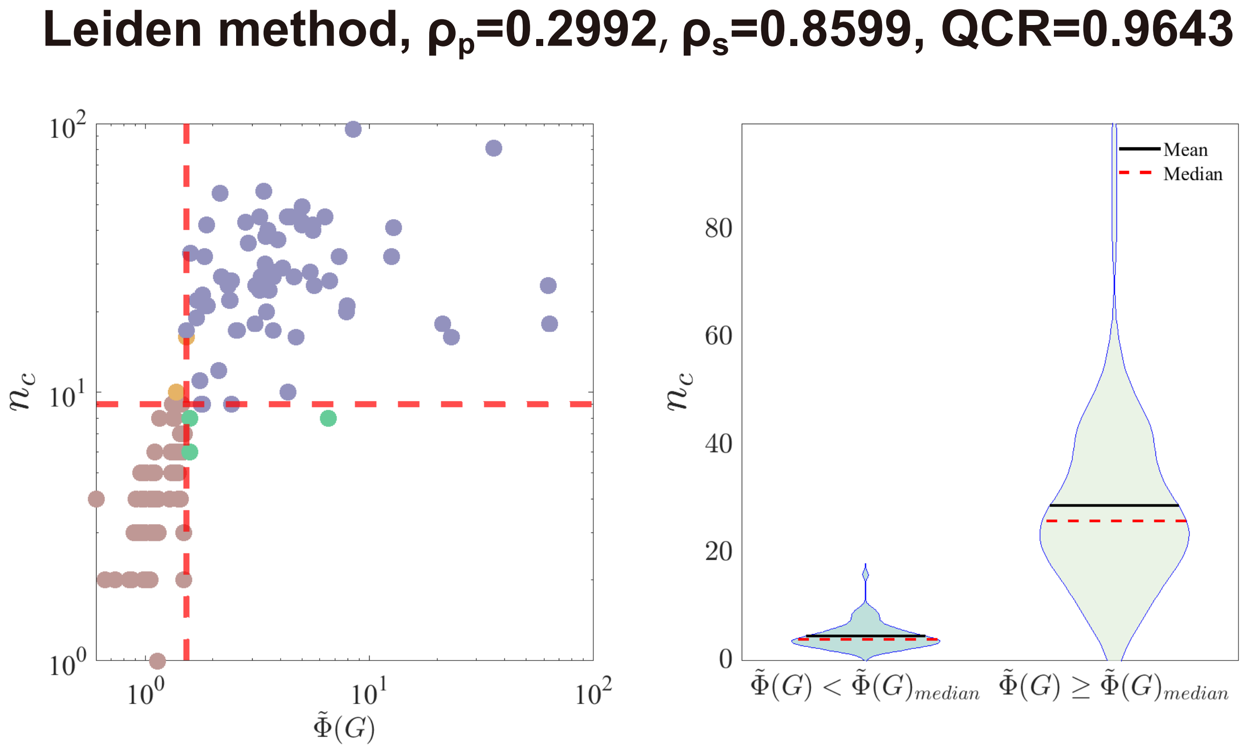

Fig. S3. Correlations between the relative path multiplicity and community number detected by the Leiden method in 140 real-world networks. The metadata and results for all 140 networks are provided in Table S1. Each panel presents a scatter plot on the left, illustrating the correlation between the relative path multiplicity and community number (detected by Leiden method) based on the given metric and their RPMI values. The networks are categorized into four quadrants, divided by the median values of community number n_c and the RPMI $\tilde{\Phi}(G)$. The right-hand violin plots show the distribution, mean and median values of community number, which are divided into two parts based on the median value of the RPMI. If there are more overlap between two violin subplots, it suggests a weaker correlation between community number and the RPMI. All community detection packages/functions and parameter settings used in this study, together with brief documentation, are available at https://github.com/gituserbnu/path-multiplicity.git.

Label Propagation method, ρ_p=0.1913, ρ_s=0.8450, QCR=0.9357

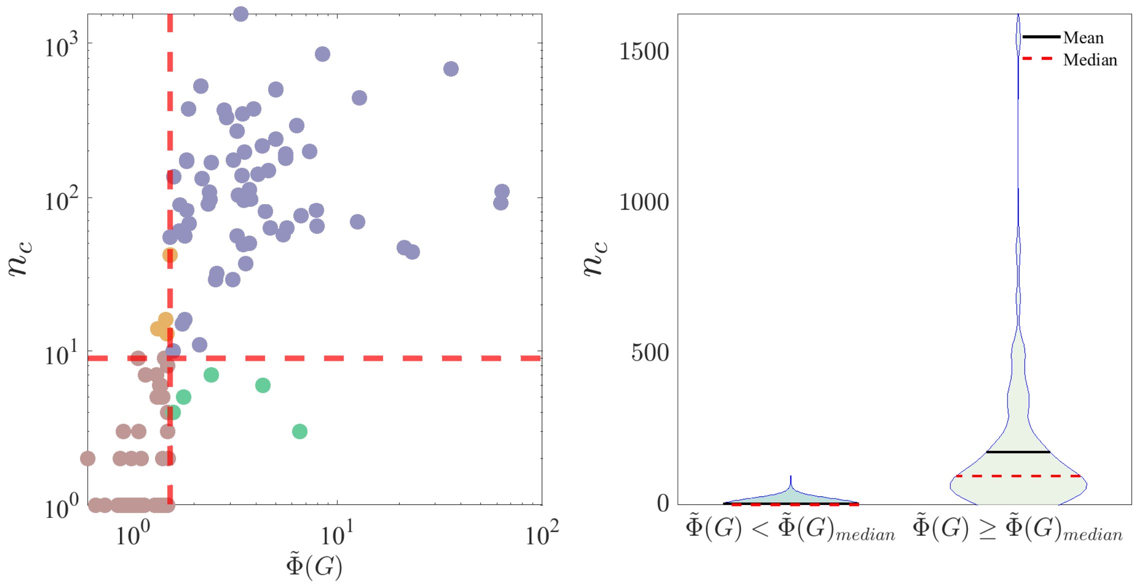

Fig. S4. Correlations between the relative path multiplicity and community number detected by the Label Propagation method in 140 real-world networks. The metadata and results for all 140 networks are provided in Table S1. Each panel presents a scatter plot on the left, illustrating the correlation between the relative path multiplicity and community number (detected by Label Propagation method) based on the given metric and their RPMI values. The networks are categorized into four quadrants, divided by the median values of community number n_c and the RPMI $\tilde{\Phi}(G)$. The right-hand violin plots show the distribution, mean and median values of community number, which are divided into two parts based on the median value of the RPMI. If there are more overlap between two violin subplots, it suggests a weaker correlation between community number and the RPMI. All community detection packages/functions and parameter settings used in this study, together with brief documentation, are available at https://github.com/gituserbnu/path-multiplicity.git.

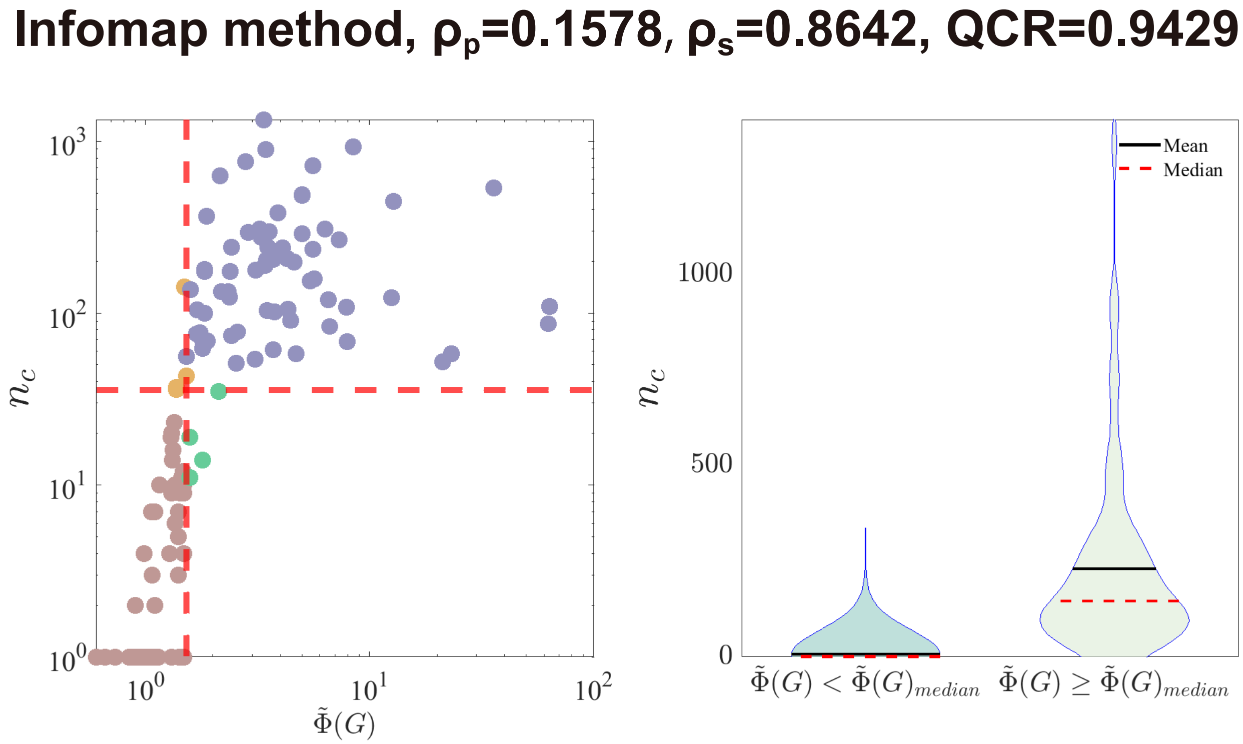

Fig. S5. Correlations between the relative path multiplicity and community number detected by the Infomap method in 140 real-world networks. The metadata and results for all 140 networks are provided in Table S1. Each panel presents a scatter plot on the left, illustrating the correlation between the relative path multiplicity and community number (detected by Infomap method) based on the given metric and their RPMI values. The networks are categorized into four quadrants, divided by the median values of community number n_c and the RPMI $\tilde{\Phi}(G)$. The right-hand violin plots show the distribution, mean and median values of community number, which are divided into two parts based on the median value of the RPMI. If there are more overlap between two violin subplots, it suggests a weaker correlation between community number and the RPMI. All community detection packages/functions and parameter settings used in this study, together with brief documentation, are available at https://github.com/gituserbnu/path-multiplicity.git.

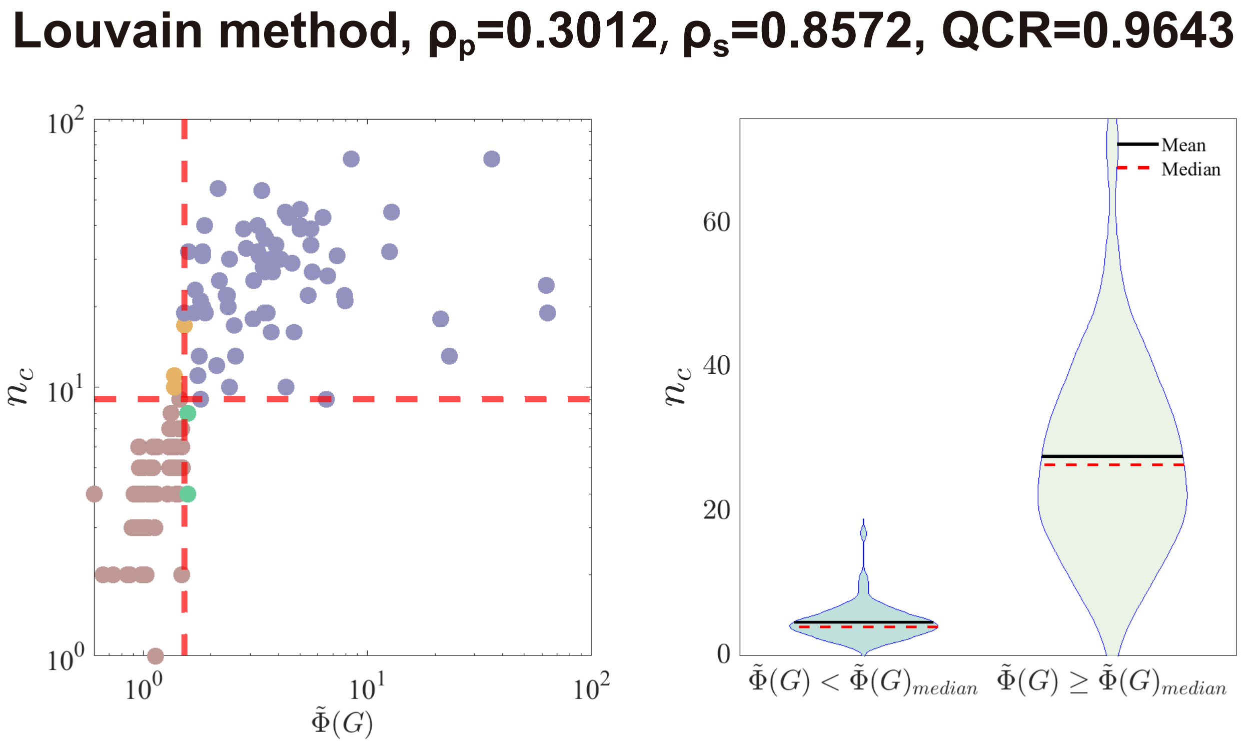

Fig. S6. Correlations between the relative path multiplicity and community number detected by the Louvain method in 140 real-world networks. The metadata and results for all 140 networks are provided in Table S1. Each panel presents a scatter plot on the left, illustrating the correlation between the relative path multiplicity and community number (detected by Louvain method) based on the given metric and their RPMI values. The networks are categorized into four quadrants, divided by the median values of community number n_c and the RPMI $\tilde{\Phi}(G)$. The right-hand violin plots show the distribution, mean and median values of community number, which are divided into two parts based on the median value of the RPMI. If there are more overlap between two violin subplots, it suggests a weaker correlation between community number and the RPMI. All community detection packages/functions and parameter settings used in this study, together with brief documentation, are available at https://github.com/gituserbnu/path-multiplicity.git.

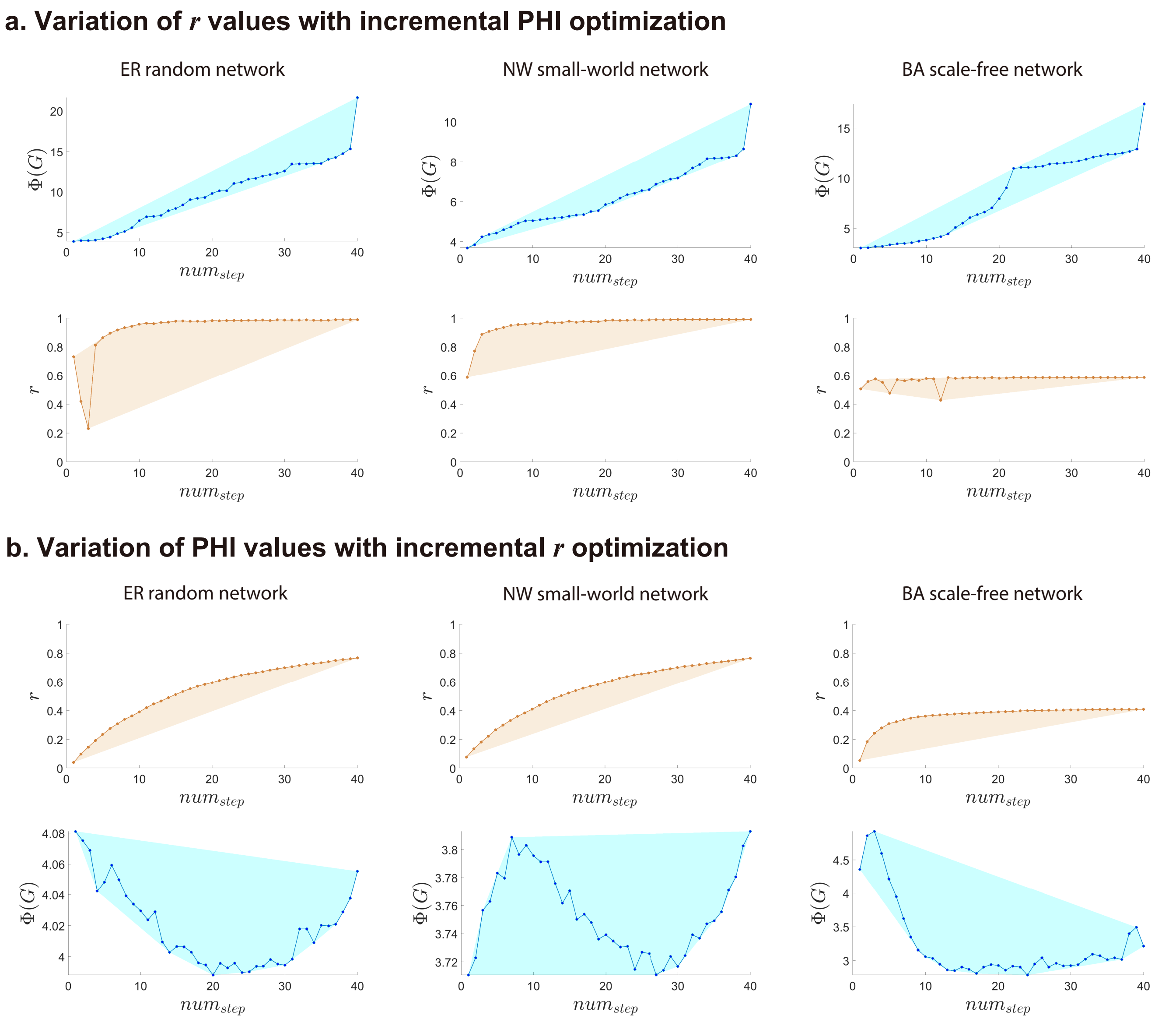

Fig. S7. Target-oriented edge rewiring in three types of model networks. **a,** Variation in the assortativity r values with incremental optimization of the PMI through target-oriented edge rewiring. As the PMI increases, the value of assortativity r exhibits a model-dependent, nonmonotonic trend. **b,** Variation in the PMI values with incremental optimization of assortativity r through target-oriented edge rewiring. In this scenario, edge rewiring is directed toward increasing r, resulting in a nonmonotonic (often U-shaped) trend in the PMI values. Shaded regions indicate simple envelopes for visual guidance, not uncertainty or confidence intervals.

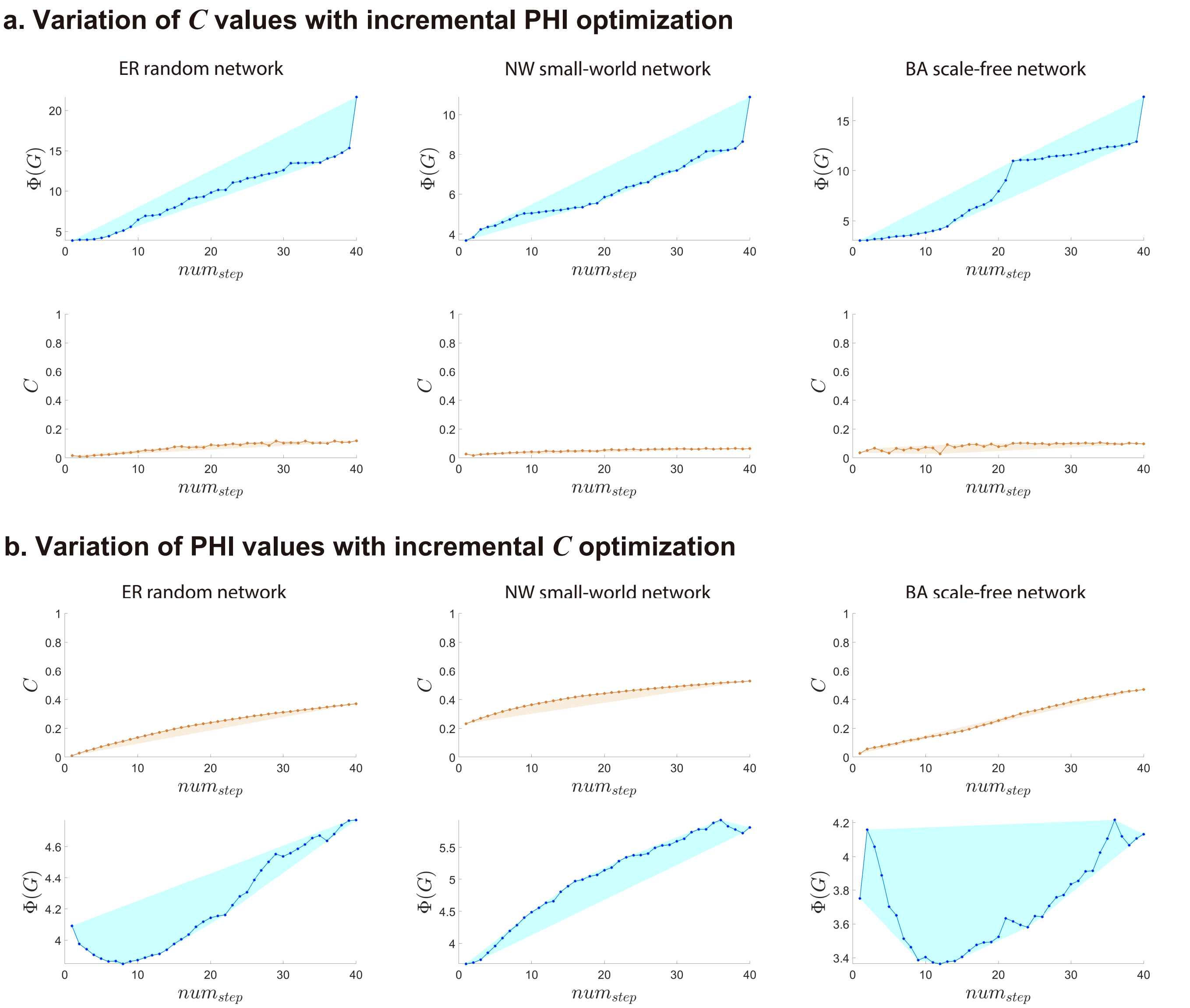

Fig. S8. Target-oriented edge rewiring in three types of model networks. **a,** Variation in the clustering coefficient C values with incremental optimization of the PMI through target-oriented edge rewiring. As the PMI increases, the value of the clustering coefficient C shows a weak, model-dependent trend consisting of small increases in ER/NW networks and near constancy in BA networks. **b,** Variation in the PMI values with incremental optimization of the clustering coefficient C through target-oriented edge rewiring. In this scenario, edge rewiring is directed toward increasing C, resulting in a nonmonotonic (often U-shaped) trend in the PMI values. Shaded regions indicate simple envelopes for visual guidance, not uncertainty or confidence intervals.

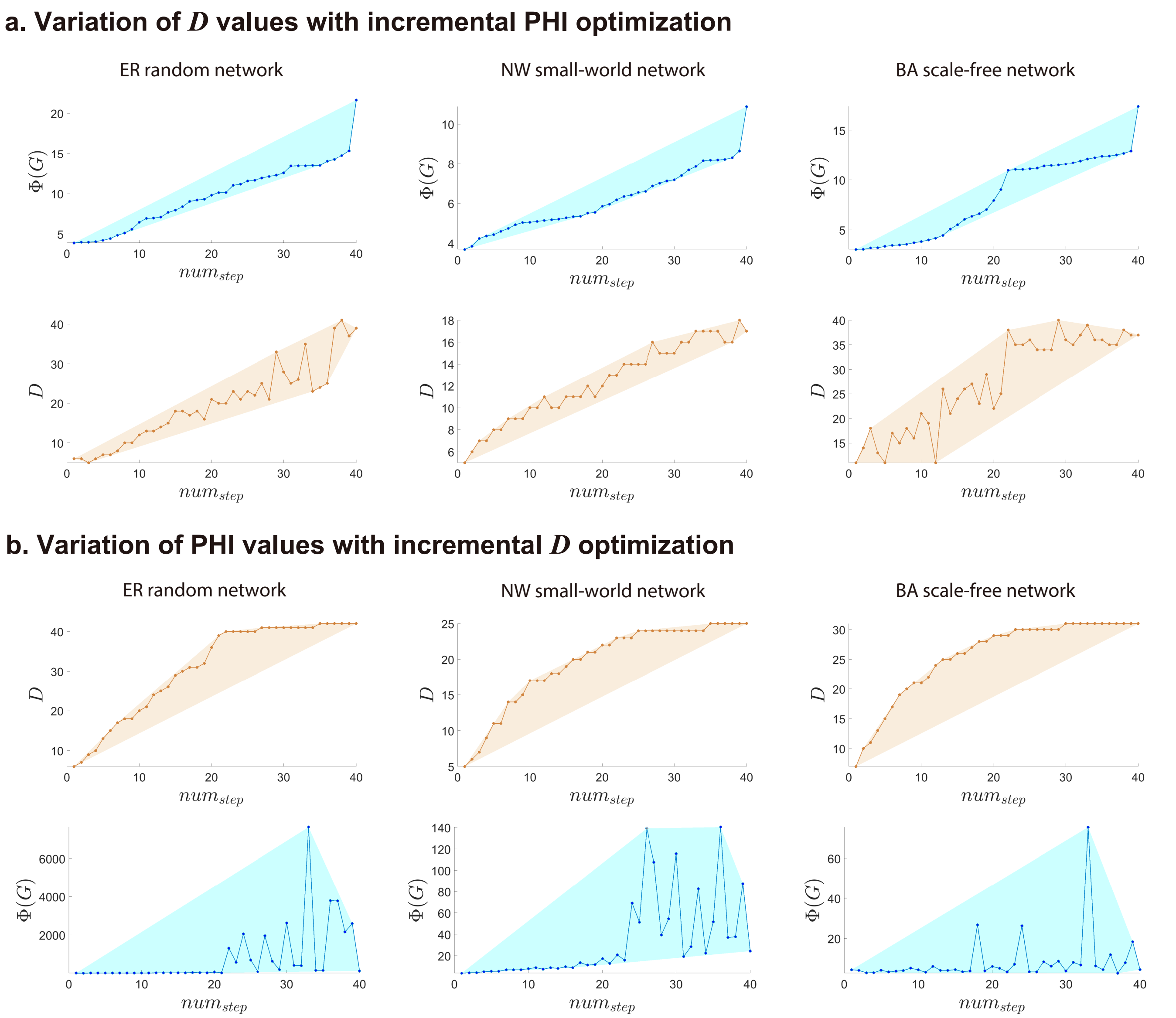

Fig. S9. Target-oriented edge rewiring in three types of model networks. **a,** Variation in the diameter D values with incremental optimization of the PMI through target-oriented edge rewiring. As the PMI increases, the value of diameter D generally increases with the model-dependent fluctuations. **b,** Variation in the PMI values with incremental optimization of the diameter D through target-oriented edge rewiring. In this scenario, edge rewiring is directed towards increasing D, resulting in a highly nonmonotonic, spike-like trend with intermittent peaks and no sustained increase in the PMI values. Shaded regions indicate simple envelopes for visual guidance, not uncertainty or confidence intervals.
